# Determination of Progestin Residues in Fish by UPLC-Q-TOF/MS Coupled with QuEChERS

**DOI:** 10.1155/2019/6426958

**Published:** 2019-05-02

**Authors:** Chunxiu Gu, Yanling Cheng, Xin Zhen, Xiaoxuan Chen, Kaowen Zhou

**Affiliations:** ^1^Biochemical Engineering College, Beijing Union University, Beijing 100023, China; ^2^Beijing Key Laboratory of Biomass Waste Resource Utilization, Beijing 100023, China

## Abstract

A novel method was developed for simultaneous determination of 10 progestin residues in fish by ultra-performance liquid chromatography-quadrupole-time-of-flight mass spectrometry (UPLC-Q-TOF/MS) combined with a modified quick, easy, cheap, effective, rugged, and safe (QuEChERS) method. The homogenized samples were dispersed by water, extracted with acetonitrile, and then purified by QuEChERS reagent. The concentrated analytes were detected by UPLC-Q-TOF/MS. High linearities (*R*^2^ > 0.995) and recoveries (85.71–117.08%) at three spiked levels (5, 10, and 20 ng/g) and low relative standard deviation values (<8.83%, *n* = 7) and limits of detection (0.23–0.66 ng/g) were obtained. This method is simple, rapid, reliable, sensitive, and efficient and can be used for monitoring of progestin residues in fish. This method provides a strong guarantee to deal with food emergencies for the laboratory, provides technical support for the screening and quantitative detection of progesterone in fish, and provides technical support for the food safety of aquatic products.

## 1. Introduction

Hormone is a kind of substance produced by organisms to regulate metabolism or physiological functions of the body [1–3]. It is used to promote animal growth and improve the protein conversion rate in aquaculture to achieve the goal of greatly improving economic benefits of animal breeding. Studies have shown that progestin, a class of hormones, can enter animal tissues through feedstuffs and be ingested indirectly by the human body. Residual progestin can interfere with the balance of natural hormones in the human body, cause women to have similar reactions to early pregnancy, breast swelling, and irregular bleeding, affect liver function, cause neonatal malformations, and even cause cancer [4]. Therefore, it is necessary to develop a fast and accurate method for detecting progesterone in food.

Various methods were applied to determine progestin, including gas chromatography mass spectrometry (GC-MS) [5], high-performance liquid chromatography tandem mass spectrometry (HPLC-MS/MS) [6–9], fluorescent immunoassay [10, 11], and electrochemistry [12, 13]. Generally, derivatization steps are required in GC-MS analysis, which may be time consuming and lead to variety limitation, because most derivative processes are selective for limited groups of target analytes [14]. Compared with GC-MS, HPLC-MS/MS is supposed to show high sensitivity and specificity without additional derivatization and plays a vital role in determining a wide spectrum of compounds. Time-of-flight mass spectrometry (TOF-MS) can provide structural information of unknown compounds because of its high resolution and high-quality number accuracy [15–21]. It is possible to screen and detect drugs in unknown samples by matching characteristic fragments with accurate mass number.

In this work, an improved QuEChERS rapid pretreatment technique combined with UPLC-Q-TOF was used firstly to establish a method for simultaneous determination of 10 progestins in freshwater fish. The precise quality database of aquatic products can provide an efficient and convenient method for the determination of hormones in aquatic products and control the safety risk level of edible aquatic products.

## 2. Materials and Methods

### 2.1. Chemical Substances and Apparatus

Methanol (≥99.9%) and acetonitrile (≥99.9%) were purchased from Sigma-Aldrich (St. Louis, USA). All reagents used were of analytical grade. Anhydrous magnesium sulfate (MgSO_4_), sodium chloride (NaCl), primary secondary amine (PSA), and alumina-N were purchased from Macklin Biochemical Co., Ltd. (Shanghai, China). High-purity (least 98% purity) analytical standards were used. Norethisterone, 17*α*-hydroxyprogesterone, D-methylnorethindrone, 17*α*-hydroxyprogesterone acetate, megestrol, medroxyprogesterone, chlorprogestone acetate, medroxyprogesterone acetate, melengestrol acetate, and progesterone were purchased from Sigma-Aldrich (St. Louis, USA).

Individual stock standard solutions (1000 mg/L) of each compound were prepared using methanol and stored in the refrigerator at −20°C. The mixed standard solution was prepared by mixing the 10 individual stock standard solutions (100 *μ*L for each) and diluting with methanol to 10 mL in a volumetric flask, and the final concentration of each progestin was 10 mg/L. The mixed standard solutions prepared were stored at 4°C.

Acquity ultra-performance liquid chromatography (Waters USA); Synapt G2-Si quadrupole time-of-flight mass spectrometry (Waters USA); GR22GIII high-speed refrigerated centrifuge (HITACHI Japan); N-EVAPTM 112 nitrogen blowing apparatus (Organomation, USA); and KQ-500DE numerical control ultrasonic cleaner (Kunshan, China).

### 2.2. Sample Preparation

Fresh fish samples, i.e., grass carp, silver carp, bream, crucian carp, and catfish, were processed, homogenized, and stored at −20°C. Among these species, grass carp was used as the blank sample matrix for the method development and validation. The 10 progestins were not detected in the grass carp sample.

Two grams of crushed homogeneous fish meat was accurately weighed and put in a 50 mL spiral cap centrifugal tube, 2 mL water was added and stirred for 1 minute on the vortex oscillator, 10 mL acetonitrile was added, stirred for 1 minute, and placed on the ultrasonic oscillator for 20 minutes, 2.0 g anhydrous magnesium sulfate and 1.0 g sodium chloride were added, and quickly mixed, stirred, and put it in the ice water bath. The mixture was centrifuged at 5000 r/min for 5 min.

The supernatant was removed 8 mL and put into another 15 mL screw cap centrifugal tube. The centrifugal tube was loaded with QuEChERS purification powder (0.5 g anhydrous magnesium sulfate, 0.5 g neutral alumina, and 0.2 g PSA). Centrifugal tubes were swirled 1 min and centrifuged at 5000 r/min for 5 min. The supernatant was accurately removed, dried at 50°C on a nitrogen blower, and dissolved by 1.0 mL methanol-water (1 : 1). After passing through 0.22 *μ*m microporous membrane, the filtrate was ready for use.

### 2.3. Analysis Conditions

The analytes were separated on a Waters Acquity BEH C18 column (100 mm × 2.1 mm i.d., 1.7 *μ*m). The mobile phase was 0.1% formic acid solution (A) and methanol (B). The optimized separation conditions were as follows: the column oven temperature was kept at 40°C and the sample injection volume was 10 *μ*L. The elution gradient program was performed as follows: 0–2 min, 60% A; 2–6 min, 40% A; 6–6.5 min, 20% A, 6.5–7.1 min, 0% A; and 7.1–10 min, 60% A. The flow rate of the mobile phase was 0.3 mL/min.

The quantitative analysis of the targeted compounds was performed with the full scan (MSe) mode. Nitrogen gas was used as the drying gas, and argon gas was used as collision gas. The ionization source conditions were used as follows: the flow rate and the temperature of the nitrogen gas was set at 800 L/h and 400°C, the cone voltage was 20 V, the capillary voltage was 2000 V, ion source temperature was 100°C, collision low energy was 6 V, and high energy gradient was 10–50 eV.

### 2.4. Method Validation

To validate the developed method, limits of detection (LODs), limits of quantification (LOQs), linearity, accuracy, and precision were investigated. The LOD and LOQ values were evaluated to be 3 and 10 times the standard deviation of 7 replicate runs for a fish sample spiked to a concentration level of 1.0 ng/g. Linearity was assessed using the coefficient of determination (*R*^2^), which was obtained after constructing seven-point calibration curves. Accuracy was evaluated by the recovery of each target analyte at low, medium, and high concentrations (5, 10, and 20 ng/g) in fish samples. The precision was investigated by the relative standard deviation (RSD) of the analytical values obtained when the sample was repeatedly analyzed for 7 times (*n* = 7).

## 3. Results and Discussion

### 3.1. Optimization of Parameters in QuECHERS

The purification effect of C18 adsorbent and PSA was compared. The results showed that C18 adsorbent could adsorb the tested substance with low recovery. The adsorption efficiency of anhydrous magnesium sulfate and neutral alumina is not ideal when the mass of anhydrous magnesium sulfate and neutral alumina is relatively low. Therefore, the ratio of 0.5 g anhydrous magnesium sulfate, 0.5 g neutral alumina, and 0.2 g PSA is adopted. As an extracting solvent, acetonitrile can denaturate and precipitate the protein in the sample, thus removing the influence of the protein on the sample analysis. Anhydrous magnesium sulfate can remove moisture from the sample. Sodium chloride promotes the separation of residual water in the sample solution from acetonitrile and transfers all the target substances to the acetonitrile layer. Neutral alumina can remove the fat in the sample solution, and PSA can remove the carbohydrates and fatty acids in the sample matrix. After the above treatment, the clarified sample concentrate can be obtained, which is suitable for mass spectrometry analysis.

### 3.2. Optimization of Conditions for UPLC

This experiment compared the separation effects of three lengths 50 mm, 100 mm, and 150 mm of BEH C18. It is difficult to separate all the tested substances with a 50 mm column. A 150 mm column can completely separate the tested substances, but the separation time is longer than 10 minutes. A 100 mm column can separate the tested substances in 6 minutes, so a 100 mm column is selected.

### 3.3. Optimization of Conditions for TOF/MS

**Figure 1:**
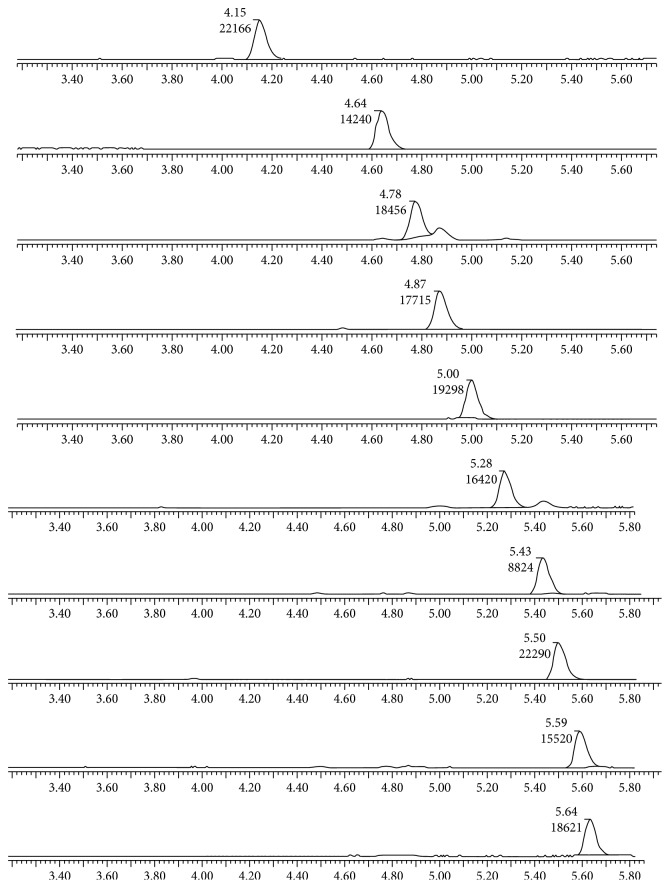
TOF mass spectrogram of 10 progestin standard solutions.

The low-energy channel acts as the first-order mass spectrometry, and the high-energy channel is used as the second-order mass spectrometry. The quasimolecular ion peaks of ten progestins were formed by the first-order mass spectrometry, and two daughter ions with high abundance were selected by the second-order mass spectrometry under the above conditions. The chromatographic retention time, quasimolecular ion peaks, and secondary ions of the compounds were used as identification characteristics. The optimized results are shown in Table 1.  Figure 1 is the TOF mass spectra of 10 progesterone matrix standard solutions.

### 3.4. Method Validation

Because of the complexity of the sample matrix, matrix effect exists in sample matrix, which can inhibit mass spectrometry signal. Standard solution can be diluted by matrix blank solution to eliminate matrix interference. Taking the concentration of hormone as abscissa and the peak area of hormone as ordinate, the standard curve was drawn, and the linear equation and correlation coefficient were obtained. The linear range of the standard curve is 10–500 ng/mL. As can be seen from Table 2, good linear determination coefficients (*R*^2^ > 0.995) of the calibration curves were obtained for all the analytes, and low LOD and LOQ values were achieved in range of 0.23–0.66 ng/g and 0.77–2.20 ng/g for selected analytes, respectively.

The blank grass carp meat was used as the substrate, and 5 ng/g, 10 ng/g, and 20 ng/g were added to the weighted substrate, respectively. Seven parallel tests were conducted at each level to determine the recovery and precision of the standard addition. The results are shown in Table 3. The recovery rate is 85.71–117.08%, and the relative standard deviation is 2.66–8.83%. At the same time, the quality control sample recovery rate and other indicators all reach the analysis requirements, indicating that the results are accurate and reliable.

## 4. Conclusions

The improved QuEChERS method was used for sample pretreatment and ultra-performance liquid chromatography quadrupole time-of-flight mass spectrometry (UPLC-Q-TOF-MS) for the determination of 10 kinds of progesterone. The method was rapid and stable and could be used for the simultaneous determination of 10 kinds of progesterone in fish meat.

This method provides a strong guarantee to deal with food emergencies for the laboratory, provides technical support for the screening and quantitative detection of progesterone in freshwater fish, and provides technical support for the food safety of aquatic products.

## Figures and Tables

**Table 1:** TOF parameters of 10 progestins.

Compounds	Formula	Exact mass	Mother ions (m/z)	Daughter ions (m/z)	RT (min)
Norethisterone	C_20_H_26_O_2_	298.1933	299.1981	231.1714, 281.1876	4.15
17*α*-hydroxyprogesterone	C_21_H_30_O_3_	330.2195	331.2245	313.2140, 295.200	4.64
D-methylnorethindrone	C_21_H_28_O_2_	312.2089	313.2142	245.1871, 295.2029	4.76
17*α*-hydroxyprogesterone acetate	C_23_H_32_O_4_	372.2301	373.2391	313.2151, 271.2095	4.87
Megestrol	C_22_H_30_O_3_	342.2195	343.2242	325.2208, 267.1788	5.00
Medroxyprogesterone	C_22_H_32_O_3_	344.2351	345.2393	123.0765, 327.2294	5.28
Chlorprogestone acetate	C_23_H_29_ClO_4_	404.1754	405.1806	309.1852, 267.1704	5.43
Medroxyprogesterone acetate	C_24_H_34_O_4_	386.2454	387.2521	327.2306, 123.0820	5.50
Melengestrol acetate	C_25_H_32_O_4_	396.2301	397.2343	279.1719, 337.2200	5.59
Progesterone	C_21_H_30_O_2_	314.2246	315.2287	109.0609, 297.2179	5.64

**Table 2:** Regression equations, correlation coefficients (*R*^2^), LOD, and LOQ of 10 progestins.

Compounds	Linear equations	*R* ^2^	LOD (ng/g)	LOQ (ng/g)
Norethisterone	*y* = 184.1*x* + 2165	0.995	0.23	0.77
17*α*-hydroxyprogesterone	*y* = 135.3*x* + 439.8	0.999	0403	1.77
D-methylnorethindrone	*y* = 156.6*x* + 1425	0.995	0.52	1.72
17*α*-hydroxyprogesterone acetate	*y* = 152.3*x* + 1707	0.997	0.34	1.12
Megestrol	*y* = 185.8*x* + 418.2	0.999	0.66	2.20
Medroxyprogesterone	*y* = 158.9*x* + 439.7	0.999	0.27	0.91
Chlorprogestone acetate	*y* = 77.1*x* + 698	0.997	0.32	1.05
Medroxyprogesterone acetate	*y* = 198.7*x* + 1468	0.998	0.37	1.22
Melengestrol acetate	*y* = 137.3*x* + 1113	0.998	0.52	1.73
Progesterone	*y* = 168.9*x* + 1266	0.999	0.28	0.92

**Table 3:** Recoveries and relative standard deviations (%) of 10 progestins in fish (*n* = 7).

Spiked level compounds	5 ng/g		10 ng/g		20 ng/g	
	Recoveries	RSDs	Recoveries	RSDs	Recoveries	RSDs
Norethisterone	88.55	5.49	85.85	4.12	87.97	5.25
17*α*-hydroxyprogesterone	88.15	5.02	85.71	6.81	102.43	7.95
D-methylnorethindrone	105.44	5.68	88.40	4.30	90.16	6.48
17*α*-hydroxyprogesterone acetate	91.73	3.83	95.71	4.01	108.96	6.18
Megestrol	88.66	7.60	86.58	6.88	87.79	6.60
Medroxyprogesterone	104.84	4.69	103.81	3.40	89.77	6.30
Chlorprogestone acetate	96.42	7.95	109.65	2.66	97.18	7.59
Medroxyprogesterone acetate	87.97	8.30	91.24	7.31	107.59	6.33
Melengestrol acetate	89.56	8.59	99.97	7.22	91.40	8.83
Progesterone	106.36	8.28	91.58	4.63	117.08	7.76

## Data Availability

The data used to support the findings of this study are included within the article.
